# Are international differences in breast cancer survival between Australia and the UK present amongst both screen‐detected women and non‐screen‐detected women? survival estimates for women diagnosed in West Midlands and New South Wales 1997–2006

**DOI:** 10.1002/ijc.29984

**Published:** 2016-02-23

**Authors:** Laura M. Woods, Bernard Rachet, Dianne L. O'Connell, Gill Lawrence, Michel P. Coleman

**Affiliations:** ^1^ Cancer Research UK Cancer Survival GroupDepartment of Non‐Communicable Disease Epidemiology, London School of Hygiene and Tropical MedicineLondonWC1E 7HT; ^2^ Cancer Research DivisionCancer Council NSWNSW 1340Australia; ^3^ Breast Cancer Audit Consultant and Former DirectorWest Midlands Cancer Intelligence Unit, Public Health Building, University of BirminghamBirminghamB15 2TT

**Keywords:** breast cancer, net survival, excess mortality, UK, Australia, New South Wales, West Midlands, cancer screening, mammography

## Abstract

We examined survival in screened‐detected and non‐screen‐detected women diagnosed in the West Midlands (UK) and New South Wales (Australia) in order to evaluate whether international differences in survival are related to early diagnosis, or to other factors relating to the healthcare women receive. Data for women aged 50 − 65 years who had been eligible for screening from 50 years were examined. Data for 5,628 women in West Midlands and 6,396 women in New South Wales were linked to screening service records (mean age at diagnosis 53.7 years). We estimated net survival and modelled the excess hazard ratio of breast cancer death by screening status. Survival was lower for women in the West Midlands than in New South Wales (5‐year net survival 90.9% [95% CI 89.9%−91.7%] compared with 93.4% [95% CI 92.6%‐94.1%], respectively). The difference was greater between the two populations of non‐screen‐detected women (4.9%) compared to between screen‐detected women, (1.8% after adjustment for lead‐time and over‐diagnosis). The adjusted excess hazard ratio of breast cancer death for West Midlands compared with New South Wales was greater in the non‐screen‐detected group (EHR 2.00, 95% CI 1.70 − 2.31) but not significantly different to that for women whose cancer had been screen‐detected (EHR 1.72, 95% CI 0.87 − 2.56). In this study more than one in three breast cancer deaths in the West Midlands would have been avoided if survival had been the same as in New South Wales. The possibility that women in the UK receive poorer treatment is an important potential explanation which should be examined with care.

## Abstract 2

**What's new?**

Breast cancer patients in Australia are known to have higher survival than those in the UK. In this study, the authors found that these international differences in survival persist in both screen‐detected and non‐screen‐detected groups, even after adjustment for both lead‐time bias and over‐diagnosis. These results suggest that it is essential that the mechanisms underlying these differences be understood, including potential variations in effective treatment between the two regions.

We have previously shown a difference of 6% in 5‐year breast cancer survival between Australia and England for women in the target age group for screening and diagnosed during the period 1996 − 1999.^1^ Examining survival by screening status has the potential to shed further light on whether international differences are more likely to be due to tumour or patient factors or to other factors relating to the healthcare women receive. We have previously identified these as possible explanations for socioeconomic differences,^2^ but they also may explain international variations in survival.^1^

The trials that led to the implementation of mammographic screening worldwide were evaluated by examining the reduction in breast cancer mortality amongst the populations of women screened.^3^ In this context, a reduction in the number of breast cancer deaths in the screened population can be interpreted as the number of cancer deaths avoided or deferred by the intervention. This outcome is helpful in evaluating the public health impact and economic value of the screening programme as a whole.

Other studies have examined the impact of mammographic screening upon individual patient survival. Analyses of survival include examinations of interval cancers^4^, ^5^ (cancers diagnosed following a normal mammogram but prior to the next screening invitation), comparisons of women in dichotomous groups (attenders vs. never‐attenders^6^ and those with screen‐detected vs. non‐screen‐detected cancers^7^) and spatial analyses.^8^ A review conducted in the UK in 2003^9^ concluded that a better understanding of the effect of screen‐detection required more detailed data. In particular, the review identified the importance of linkage of mortality data to screening invitations so that the outcome for tumours diagnosed after the introduction of screening might be examined.

Examination of survival by screening status enables us to establish, at the population level, the survival benefit afforded to women whose cancers were screen‐detected compared to women whose cancers were detected symptomatically. The disadvantage of this approach is that it is susceptible to lead‐time bias and to over‐diagnosis. Lead‐time is the additional observation time credited to women who are screen‐detected by virtue of the fact that they are asymptomatic. Breast tumours considered to be “over‐diagnosed” are those detected by screening mammography but which would not have been diagnosed during the patient's lifetime in the absence of screening.^10^ These biases together lead to apparently better survival, even if the actual time of death is not deferred. This skews estimates of survival in favour of screening, resulting in statistics which appear to show a survival advantage amongst women who have been screened, even when none might exist. Recently, methodological advances have been made into ways to account for lead‐time bias in the analysis of survival so that the underlying differences in survival can be assessed. This involves correcting the observed survival time to account for the additional follow‐up observed in the cohort as a result of screen‐detection.^11^

In this article, we examine net survival for breast cancer in screen‐detected and non‐screen‐detected women diagnosed in the West Midlands (UK) and New South Wales (Australia), applying a correction for lead‐time bias and over‐diagnosis. We use the results to discuss the extent to which the international differences in breast cancer survival between the UK and Australia may be explained by tumour or patient factors or to other factors relating to the healthcare women receive.

## Materials

**Figure 1:**
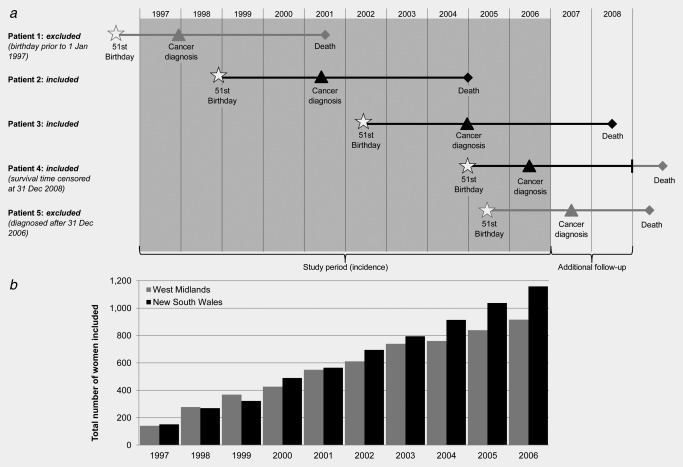
(a) Schematic diagram of women eligible for the study alongside (*b*) a histogram showing the total number of women included in New South Wales (Australia) and the West Midlands (UK) by year of diagnosis (1997–2006).

The cohort of interest consisted of women who were invited to attend for screening mammography in a fully‐functioning, mature screening programme during a defined calendar period. Women diagnosed with a primary invasive breast cancer at ages 50–65 years during the period 1 January 1997 to 31 December 2006 and aged 51 years or younger on 1 January 1997 were considered eligible (Fig. 1). We thus excluded women who were first invited to be screened at ages over 50 years, as well as women invited during the years when the screening programme was being established and expanded. We excluded women aged over 65 years at diagnosis because the target age for screening was up to age 64 years in the UK during this period. The eligibility criteria resulted in a cohort which built up over time (median month of diagnosis August 2003 in West Midlands and November 2003 in New South Wales). All women were followed up to 31 December 2008 (at least 2 years following diagnosis). Data were obtained from the West Midlands Office of the English National Cancer Registration Service (WMNCRS, England) and the New South Wales Central Cancer Registry (NSWCCR, Australia). These two registries cover populations of 5.6 and 6.9 million, respectively.^12^, ^13^

Information was obtained from each cancer registry on each woman's age at diagnosis (completed years), the month and year of their diagnosis and death (if dead), the sub‐site, grade, histology and behaviour of the tumour, and all information pertaining to the extent of disease at diagnosis (stage). Staging information for cases in the West Midlands was recoded according to the rules used by the New South Wales Central Cancer Registry: localised (confined to the organ of origin), regional (spread to adjacent muscle, organ, fat, connective tissue or regional lymph nodes), distant (distant metastasis) and unknown stage.

The cancer registry data were linked to the population‐based mammographic screening service records in each locality to establish each woman's screening status at diagnosis (the National Health Service Breast Screening Programme for the West Midlands and BreastScreen NSW for New South Wales). We defined four categories for the screening status at diagnosis: (1) women whose cancer was detected at a routine screen, (2) women who presented with cancer following a negative screen but before being invited to their next routine screen (interval cancers), (3) women who presented with cancer after at least one negative screen but who had not attended their most recent appointment (lapsed attenders), and (4) women who presented with cancer who had never attended screening. We also compared women in the screen‐detected group (Category 1) to all those with non‐screen‐detected cancer (Categories 2, 3 and 4). This broadly corresponded to comparing those with asymptomatic disease identified via routine screening to women presenting with symptomatic disease.

## Methods

### Net survival estimation

Net survival is defined as the survival from the disease of interest. It is derived by adjusting the overall survival in the patient group for their expected survival in the absence of the disease. We estimated net survival using the non‐parametric Pohar‐Perme estimator,^14^ which has been implemented in Stata.^15^ The Pohar‐Perme estimator is an unbiased estimator of net survival with respect to informative censoring (defined as the tendency for the estimates to reflect the survival of patients with lowest expected mortality as time since diagnosis increases) for population‐based data.^16^, ^17^

We estimated expected survival from region‐specific life tables provided by the Office for National Statistics for England and Wales and the Australian Bureau of Statistics^1^ for each calendar year of follow‐up.

### Adjustment for lead‐time and over‐diagnosis

**Figure 2:**
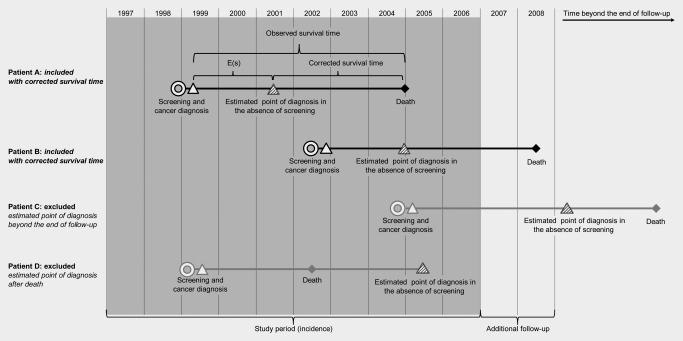
Schematic diagram demonstrating the exclusion of women in order to adjust for lead‐time bias and over‐diagnosis.

To account for the potential effect of lead‐time bias, we calculated additional survival time due to screening, E(s), for the screen‐detected group, as proposed by Duffy *et al*.^11^ and assuming a mean sojourn time (time from carcinogenesis to symptomatic cancer in the absence of screening) in both regions of 4 years. We applied 10 separate simulations to obtain a range of possible values, *E(s)_1_*, *E(s)_2_* … *E(s)_10_*, by assuming that survival times were exponentially distributed with a mean of E(s). Values of E(s) were subtracted from observed survival time in order to obtain corrected survival time (Fig. 2, Patients A and B).

We considered tumours to be over‐diagnosed if they would not have been detected symptomatically during the study period or during the predicted lifetime of the patient. To account for over‐diagnosis we excluded tumours in instances where the value of *E(s)_1_*, *E(s)_2_* … *E(s)_10_* exceeded the woman's actual observed survival time, either because the predicted date of diagnosis was after 31st December 2008, or before her death. (Fig. 2, Patients C and D).

**Figure 3:**
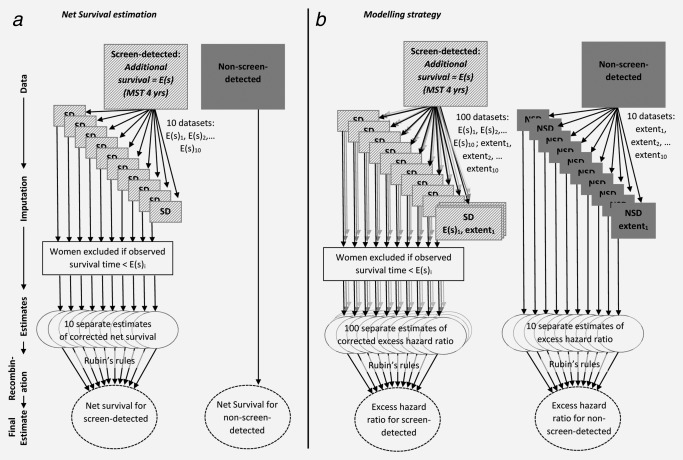
Schematic diagram illustrating (*a*) net survival estimation correcting for lead‐time bias and over‐diagnosis and (*b*) the modelling strategy taking into account missing values for extent of disease.

We used the corrected survival times to estimate non‐parametric net survival for each of these ten separate data sets for the screen‐detected group. We used the rules established by Rubin^18^ for the re‐combination of estimates in a multiple‐imputation setting to derive an overall estimate of net survival and its variance, adjusted for lead‐time bias and over‐diagnosis (Fig. 3 *a*).

### Missing data

Data on extent of disease were missing for 8.9% of women diagnosed in West Midlands and 5.3% of those diagnosed in New South Wales. We used a 10‐fold hot‐deck approach to take account of these missing values for extent of disease. The hot‐deck approach involves identifying ‘donor groups’ for each woman with missing information on extent of disease. The donor group for each woman comprised women diagnosed in the same period (1997–2000, 2001–2006) and region (West Midlands, New South Wales), at a similar age (2 groups: 50–53 years [prevalent screening round], 54+ years [incident screening rounds]) who had been followed for a similar amount of time (6 groups: up to 1 year, 1‐1.9 years, 2‐3.9 years, 4‐5.9 years, 6‐7.9 years and 8+ years), and with the same vital status at the end of follow‐up (dead, alive), and screening status (screen‐detected, not screen‐detected). For each woman with missing data, ten separate values of extent of disease (*extent_1_*, *extent_2_* … *extent_10_)* were obtained by randomly and independently selecting values of extent of disease from the donor group.

Combining these two procedures resulted in data sets with a set of 10 imputed values for the variable *extent_j_* for both the screen‐detected group and non‐screen‐detected group, and a set of 10 imputed values for the variable *E(s)_i_* for the screen‐detected only (where *i* = 1–10).

### Modelling

We fitted flexible non‐parametric regression models for net survival^19^ to estimate the excess hazard ratio associated with being diagnosed with breast cancer in the West Midlands compared to New South Wales. We fitted 10 models for women with screen‐detected cancer using the values *E(s)_1_ to E(s)_10_* combined with *extent_1_* and one model for the non‐screen‐detected cancer using observed survival times and values of *extent_1_*. *A priori*, we included age at diagnosis, region and extent of disease in the models. We used a reduction of 3 or more in the AIC (Akaike Information Criterion) to indicate a better fit. We examined non‐linearity of age by the inclusion of restricted cubic splines and tested for time‐varying effects for region, age at diagnosis, and extent of disease. We examined interactions between region and age, and between region and extent of disease.

For the screen‐detected group, we applied the model with the smallest number of parameters to each unique combination of *E(s)_i_* and *extent_j_* (100 separate combinations of results). For non‐screen‐detected women we refitted the model found to fit best to using values of *extent_1_* to the data for *extent_2_, extent_3_ … extent_10_* (10 sets of results).

We predicted from the final models estimates of crude mortality^20^ due to breast cancer and crude mortality due to other causes for the whole cohort. Crude mortality can be derived directly from the net survival models,^21^ and allows the mortality observed during follow‐up to be partitioned into mortality due to the cancer itself and due to other causes. Estimates of crude mortality were derived for each of the covariate patterns in the sample and a weighted average of deaths due to breast cancer across all patterns was calculated by region and screening. Estimates were derived separately for screen‐detected women and non‐screen‐detected women in West Midlands and New South Wales.

We used Rubin's rules^18^ to re‐combine the 100 separate estimates of the excess hazard ratio of breast cancer death and crude mortality from breast cancer for screen‐detected women and the 10 separate estimates for non‐screen‐detected women. This resulted in separate estimates for screen‐detected and non‐screen‐detected women of the relative change in the excess hazard of death due to breast cancer for women living in West Midlands compared to women in New South Wales, as well as the crude probability of death from breast cancer and other causes, and their associated variances. These final estimates took into account lead‐time bias and over‐diagnosis in the screen‐detected group and were also adjusted for age and extent of disease at diagnosis (Fig. 3 *b*).

The estimates of crude mortality were used to establish the number of cancer deaths that could have been avoided in the hypothetical situation in which survival was equalised between the two regions. This provides an estimate of the public health impact of survival differentials^22^ in the net survival setting.

## Results

We analysed data for 5,628 women in West Midlands (98.5% of those eligible, mean age at diagnosis 53.7 years) and 6,396 women in New South Wales (99.9% of those eligible, mean age at diagnosis 53.8 years). Those excluded were the very small number of women who were known to the registry only because breast cancer had been mentioned on their death certificate (DCOs) or because the sequence of dates provided was illogical. The proportion of tumours that were screen‐detected was greater in West Midlands (44.8% compared to 36.5%, Table 1). The majority of women were diagnosed with localised disease, (54.1% in West Midlands, 53.9% in New South Wales). Fewer than one in ten women died during follow‐up: 10.8% in West Midlands and 7.6% in New South Wales.

**Table 1:** Net survival estimates at 1 and 5 years after diagnosis by mode of presentation and extent of disease at diagnosis: women aged 50–65 (mean age 53.7 years) diagnosed with invasive breast cancer 1 January 1997–31 December 2006 and followed up to 31 December 2008 in New South Wales (Australia) and the West Midlands (UK)

	New South Wales					West Midlands				
		Deaths (% of *N*) within		Net Survival, % (CI)			Deaths (% of N) within		Net Survival^a^, % (CI)	
(a) Mode of presentation	*N* (%)	1 year	5 years	1‐year	5‐year	N (%)	1 year	5 years	1‐year	5‐year
Screen‐detected	2,335 (36.5)	11 (0.5)	54 (2.3)	99.8 (99.2,99.9)	98.5 (97.5,99.1)	2,524 (44.8)	11 (0.2)	90 (1.4)	99.9 (98.8,100.0)	97.5 (96.4,98.3)
*adjusted for lead‐time* b	*1,390 (21.7)*	*10 (0.7)*	*48 (3.5)*	*98.9 (98.3,99.5)*	*96.5 (95.2,97.9)*	*1,534 (27.3)*	*10 (0.7)*	*81 (5.3)*	*98.6 (97.9,99.3)*	*94.7 (93.2,96.2)*
Lapsed‐attender	129 (2.0)	4 (3.1)	16 (12.4)	97.2 (92.0,99.0)	86.8 (78.2,92.2)	175 (3.1)	6 (0.1)	17 (0.3)	96.9 (92.7,98.7)	89.8 (82.6,94.2)
Interval cancer	1,028 (16.1)	4 (0.4)	64 (6.2)	99.8 (98.4,100.0)	93.5 (91.3,95.2)	1,537 (27.3)	34 (0.5)	157 (2.5)	98.1 (97.2,98.7)	90.3 (88.4,92.0)
Never‐attender	2,904 (45.4)	86 (3.0)	297 (10.2)	97.3 (96.6,97.8)	89.5 (88.1,90.7)	1,392 (24.7)	97 (1.5)	280 (4.4)	93.3 (91.8,94.5)	79.8 (77.4,82.0)
All groups^c^	6,396 (100.0)	105 (1.6)	431 (6.7)	98.6 (98.3,98.9)	93.4 (92.6,94.1)	5,628 (100.0)	148 (2.3)	544 (8.5)	97.7 (97.2,98.1)	**90.9 (89.9,91.7)**

**(b)** Extent of disease at diagnosis	Non‐screen‐detected					Screen‐detected					Non‐screen‐detected					Screen‐detected				
		Deaths (% of N) within		Net Survival, % (CI)			Deaths (% of N) within		Net Survival, % (CI)			Deaths (% of N) within		Net Survival, % (CI)			**Deaths (% of N) within**		**Net Survival, % (CI)**	
	*N* (%)	1 year	5 years	1‐year	5‐year	N (%)	1 year	5 years	1‐year	5‐year	N (%)	1 year	5 years	1‐year	5‐year	N (%)	**1 year**	**5 years**	**1‐year**	**5‐year**
Localised	1,955 (48.1)	7 (0.1)	71 (4.1)	99.9 (98.8,100.0)	96.9 (95.7,97.8)	1,490 (64.1)	3 (0.1)	16 (1.1)	99.9 (99.0,100.0)	99.9 (12.5,100.0)	1,351 (44.1)	6 (0.1)	59 (4.4)	99.9 (98.4,100.0)	96.8 (95.3,97.9)	1,702 (67.1)	4 (0.1)	36 (2.1)	100.0 (100.0,116.3)	99.1 (97.4,99.7)
*adjusted for lead‐time* b	*N/A*	*N/A*	*N/A*	*N/A*	*N/A*	*875 (37.1)*	*2 (0.1)*	*10 (1.1)*	*99.7 (99.1,100.2)*	99.2* (98.0,100.5)	*N/A*	*N/A*	*N/A*	*N/A*	*N/A*	*1,008 (40.1)*	*3 (0.1)*	*27 (3.1)*	*99.4 (98.8,100.0)*	*97.3 (95.7,98.8)*
Regional	1,644 (40.1)	19 (1.1)	162 (10.1)	99.1 (98.4,99.5)	89.5 (87.6,91.1)	693 (30.1)	7 (1.1)	28 (4.1)	99.2 (98.0,99.7)	96.3 (93.9,97.8)	1,319 (42.1)	39 (3.1)	241 (18.3)	97.3 (96.3,98.1)	80.9 (78.3,83.2)	634 (25.1)	6 (1.1)	49 (8.1)	99.4 (97.9,99.8)	92.6 (89.6,94.8)
Distant	224 (6.1)	56 (3.1)	99 (6.1)	75.1 (68.9,80.3)	51.2 (43.6,58.3)	52 (2.1)	1 (2.1)	6 (12.1)	98.3 (85.8,99.8)	‐	115 (4.1)	54 (47.1)	83 (72.2)	53.1 (43.6,61.7)	19.5 (11.1,29.6)	9 (0.1)	0(0.0)	1(0.1)	‐	89.7 (44.5,98.6)
Unknown	238 (6.1)	12 (1.1)	45 (3.1)	95.2 (91.5,97.3)	79.9 (73.5,85.0)	100 (4.1)	0 (0.0)	4 (4.1)	‐	96.4 (87.0,99.0)	319 (10.1)	38 (12.1)	71 (22.3)	88.3 (84.2,91.4)	77.7 (72.2,82.2)	179 (7.1)	1 (1.1)	4 (2.1)	99.7 (84.8,100.0)	99.3 (81.9,100.0)
All stages^c^	4,061 (100.0)	94 (2.1)	377 (9.1)	97.9 (97.4,98.3)	90.4 (89.3,91.5)	2,335 (100.0)	11 (0.5)	54 (2.3)	99.8 (99.2,99.9)	98.5 (97.5,99.1)	3,104 (100.0)	137 (4.1)	454 (14.6)	95.9 (95.1,96.6)	85.5 (84.1,86.9)	2,524 (100.0)	11 (0.2)	90 (1.4)	99.9 (98.8,100.0)	97.5 (96.4,98.3)
*adjusted for lead‐time*	*N/A*	*N/A*	*N/A*	*N/A*	*N/A*	*1,390 (59.5)*	*10 (0.7)*	*48 (3.5)*	*98.9 (98.3,99.5)*	*96.5 (95.2,97.9)*	*N/A*	*N/A*	*N/A*	*N/A*	*N/A*	*1,534 (60.8)*	*10 (0.7)*	*81 (5.3)*	*98.6 (97.9,99.3)*	*94.7 (93.2,96.2)*

a Net survival estimate at the time of previous event before 1st or 5th anniversary of diagnosis. Where no estimate is given (‐) no event occurred in the first 12 months after diagnosis (1 year estimates) or between the third and fifth years after diagnosis (5 year estimates)

b Cases are excluded due to imputed follow‐up being greater than observed follow‐up (see text). Values are the mean of the 10 imputed data sets with the exception of * which is the mean of 8 estimates.

c Not adjusted for lead‐time.

**Figure 4:**
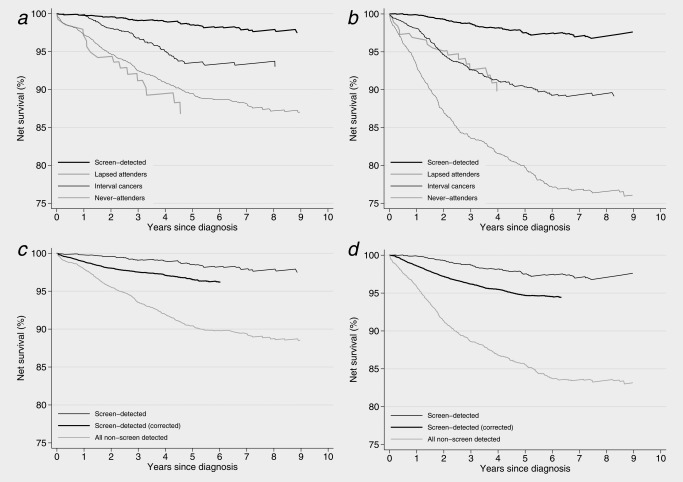
Net survival estimates for women aged 50–65 (mean age 53.7 years) diagnosed with breast cancer 1 January 1997–31 December 2006 and followed up to 31 December 2008. (*a*) by screening status, New South Wales, (*b*) by screening status, West Midlands, (*c*) screen‐detected compared to non‐screen‐detected, New South Wales, (*d*) screen‐detected compared to non‐screen‐detected, West Midlands.

Overall, net survival in the cohort was high (Table 1). Consistent with our previous findings,^1^ net survival overall was significantly lower in the West Midlands than in New South Wales (5‐year net survival 90.9% [95% CI 89.9%−91.7%] and 93.4% [95% CI 92.6%−94.1%], respectively). Women diagnosed with interval cancers in New South Wales had lower survival than screen‐detected women (5‐year net survival 93.5% compared to 98.5%), but better survival than women who had never attended screening (89.5%) and those who had attended previously but lapsed in attendance prior to diagnosis (86.8%; Table 1, Fig. 4 *a*). In West Midlands, however, the survival of women diagnosed with interval cancers was not dissimilar to that of lapsed attenders, whilst those who had never attended had the worst survival (Table 1, Fig. 4 *b*). The difference in net survival between West Midlands and New South Wales was greater among non‐screen‐detected women (4.9% five years after diagnosis) than among screen‐detected women in the two regions (1.8%; 1.0% before adjustment for lead‐time bias, Table 1).

The final models were adjusted for age and extent of disease at diagnosis. For screen‐detected women all effects (excess hazard ratios of breast cancer death) were constant over follow‐up time and followed a log‐linear form. The effect of age upon survival amongst non‐screen‐detected women was non‐linear. The effect of both age and extent of disease were found to change over follow‐up time amongst non‐screen‐detected women. The excess hazard of death from breast cancer within five years of diagnosis in the baseline model was 57% higher among women diagnosed in the West Midlands than women in New South Wales (95% CI 35%‐80%, Table 2). The baseline (age‐adjusted) disadvantage was slightly greater for women with non‐screen‐detected cancer (EHR 1.65, 95% CI 1.40 − 1.89) than for women whose cancer had been screen‐detected (EHR: 1.46, 95% CI 0.73 − 2.20). After additional adjustment for extent of disease these differentials increased (EHR 2.00, 95% CI 1.70‐2.31 in the non‐screen‐detected and 1.72, 95% CI 0.87 − 2.56 for screen‐detected cancer).

**Table 2:** Numbers of deaths, excess hazard ratios of breast cancer death and estimates of avoidable mortality within five years of diagnosis: women aged 50–65 (mean age 53.7 years) diagnosed with invasive breast cancer 1 January 1997–31 December 2006 and followed up to 31 December 2008 in New South Wales (Australia) and the West Midlands (UK)

		**Non‐screen‐detected**				**Screen‐detected**	
		**New South Wales**	**West Midlands**			**New South Wales**	**West Midlands**
							
Number of women							
Total	T	4,061 (100.0)	3,104 (100.0)			2,335 (100.0)	2,524 (100.0)
Excluded when correcting for lead‐time and over‐diagnosis	E	N/A	N/A			945 (40.5)	990 (39.2)
Included in analyses	*I = T* – *E*	4,061 (100.0)	3,104 (100.0)			1,390 (59.5)	1,534 (60.8)
Excess Hazard Ratios (EHR)							
Overall EHR, adjusted only for age (95% CI) [NSW reference]		1.57 (1.35‐1.80)					
Baseline EHR, adjusted only for age (95% CI)		1.00	1.65 (1.40‐1.89)			1.00	1.46 (0.73‐2.20)
Screening‐specific EHR, adjusted (95% CI)		1.00	2.00 (1.70‐2.31)			1.00	1.72 (0.87‐2.56)
Avoidable mortality 5 years after diagnosis							
Crude mortality due to breast cancer (%)	CM	9.5	16.0			5.6	7.9
Corresponding number of deaths due to breast cancer	*D*_actual_ = *I* * CM	388	496			77	121
If excess hazard of death due to breast cancer in West Midlands was equal to New South Wales							
Deaths due to breast cancer	*D*_equal_ = *I*_WM_ * CM_NSW_	N/A	296			N/A	85
Deaths due to breast cancer that could be avoided (% of deaths due to breast cancer)	*D*_avoid_ = *D*_actual_ − *D*_equal_	N/A	200 (40.2)			N/A	36 (29.5)

Crude mortality due to breast cancer 5 years after diagnosis was correspondingly much higher in the West Midlands. Amongst the cohort of women we examined, an estimated total of 236 deaths, 38.1% of those due to breast cancer, would have been avoided in the West Midlands had their survival been the same as those diagnosed in New South Wales; 200 (40.2%) amongst non‐screen‐detected women and 36 (29.5%) amongst those whose cancer was screen‐detected (Table 2).

## Discussion

Breast cancer survival for the women included in this study was significantly lower in West Midlands (UK) than New South Wales (Australia), which is fully consistent with our previous findings.^1^, ^23^, ^24^, ^25^, ^26^, ^27^ Our results further show the extent and persistence of this difference amongst a cohort of peri‐menopausal women who were invited for screening in a mature, fully functioning population‐based screening programme.

### Survival differences

In the West Midlands, 5‐year survival amongst women who had never attended for screening was 4.9% lower (absolute difference) than amongst the never‐attenders in New South Wales. For women whose cancer was screen‐detected, this difference was 1.7% after adjustment for lead‐time bias.

The 5‐year adjusted excess hazard ratio of breast cancer death for the non‐screened group indicates a substantial and significant survival disadvantage for West Midlands. This is striking because these estimates are adjusted for differences in age and extent of disease at diagnosis, and so one might expect survival to be much more similar. Even among screen‐detected women the survival disadvantage is distinct which is particularly striking because these are women diagnosed with asymptomatic cancers. Their tumours are predominantly localised, and as such they would almost all be treated surgically and with curative intent and have a high chance of long‐term survival.

Although the overall number of deaths is relatively modest in this cohort of cancer patients, with only 9.1% of all women dying during follow‐up, the impact of these differences is important. The increased excess hazard of breast cancer death 5 years after diagnosis in the West Midlands is double that of New South Wales amongst non‐screen‐detected women and 72% greater amongst those with a screen‐detected cancer. Overall we estimated that more than a third of the deaths attributable to breast cancer observed for women in West Midlands would have been avoided had their survival been the same as the women in New South Wales.

### Bias and artefact

Taken together, our results suggest that differences in screening practice and extent of disease at diagnosis do not explain the overall difference in survival between West Midlands and New South Wales for this age group, and that women with breast cancer in West Midlands have a higher risk of excess death from their cancer than women in New South Wales, whether they are screened or not.

### The role of ‘*de facto*’ screening

These differences in survival are likely to be in part due to the differences in the way screening is delivered in the West Midlands and in New South Wales. In the UK, the National Health Service is free at the point of delivery for the whole population and private mammography is rare. In contrast, in Australia, mammography is obtained through BreastScreen Australia but also through private radiology clinics. Mammograms conducted privately for diagnostic purposes, rather than in asymptomatic women, may be refunded via the Medicare Benefits Scheme (MBS). A substantial proportion of those conducted in private clinics is likely to constitute *de facto* screening, (regular diagnostic mammography not recorded by BreastScreen Australia), but it is unknown to what degree this occurs.^28^ This is likely to be the reason for the higher proportion of tumours in the West Midlands that were apparently screen‐detected, despite a shorter screening interval in New South Wales. It also implies that women in New South Wales whom we defined as ‘never‐attenders’ includes a sub‐group of women who had, in fact, been screened outside of the national screening programme. This interpretation is supported by the observation that a significantly larger proportion of these women classified as ‘never‐attenders’ in New South Wales were diagnosed with localised tumours (50.8% compared to 46.6% in West Midlands).

Although it is probable that we incorrectly allocated some women to the never‐attender group who were actually screen‐detected, especially in New South Wales, information on their personal characteristics and the features of their cancer would not have been compromised since these data items were collected from the Cancer Registry, rather than via the screening service. It is possible, however, that this may have biased our estimates of net survival. We therefore performed a sensitivity analysis to examine the potential for *de facto* screening to explain the difference in survival for the non‐screen‐detected group. We randomly reallocated women in New South Wales with localised disease from the non‐screen‐detected to the screen‐detected group, for selected proportions ranging from 1% to 95%, and then re‐estimated the net survival function. Over 100 iterations the five‐year net survival estimates for the non‐screen‐detected group in New South Wales became similar to those for West Midlands only when an implausible 90% of the localised cancers (43% of all non‐screen‐detected cancers, c. 1800 women) were reallocated (data not shown). This level of reallocation would require that the true proportion of cancers screen‐detected in New South Wales in the cohort was in excess of 64%, in comparison to the 36.5% actually observed (and the 44.8% observed in West Midlands, that probably reflects the order of magnitude one might expect for New South Wales, since private mammography is very rare in West Midlands). For smaller, but substantial proportions of reallocation the reduction in the survival difference was relatively small. We thus consider it very unlikely that *de facto* screening can fully explain the difference in survival between non‐screen‐detected women. This analysis also served to illustrate the robust nature of the difference for the screen‐detected group: there remained a survival advantage for New South Wales, albeit very small, even when 95% of localised (apparently symptomatic) tumours were reallocated to the screen‐detected group.

### Screening‐specific biases

We may consider whether the longer screening interval in the West Midlands compared with New South Wales (3 years versus 2 years) might contribute to these differences. Screen‐detected cancers in New South Wales could perhaps be diagnosed at an earlier stage, with better prognosis. However, the distribution by extent of disease was similar in both regions; the proportion of localised disease was in fact slightly higher in the West Midlands than in New South Wales (67.1% versus 64.1%, Table 1). A shorter screening interval will lead to detection of a greater number of slower‐growing tumours, but also greater numbers of aggressive, faster‐growing tumours, which will also be identified at an earlier stage than would otherwise be the case. In our data, the distribution of tumours by extent of disease amongst interval cancers was fairly similar in both regions (localised tumours representing 51.8% in New South Wales and 50.0% in West Midlands, Chi^2^ *p* value 0.07) This supports the interpretation that the breast cancer survival differences between New South Wales and West Midlands cannot be fully explained by the shorter screening interval in New South Wales.

We have made adjustment for lead‐time and over‐diagnosis in our analysis, and demonstrated that the survival differences observed are robust to these biases. Adjustment involved a ten‐fold simulation where both the individual survival times were shortened and the number of women included in the cohort was reduced. On average, the survival time of screen‐detected women was reduced by 1.5 years and 40% were excluded (Table 2). This latter proportion does not represent the percentage of tumours over‐diagnosed, but rather the probability that a screen‐detected cancer would not have been detected symptomatically during the period of time between the actual date of diagnosis and 31st December 2006 (the mean of which was 3.4 years). The number of tumours over‐diagnosed might be reasonably obtained by estimating the probability that the cancer would not have been detected symptomatically during the woman's remaining expected life time (the mean of which was 30.6 years).

### Other non‐causal explanations

We have previously summarised the possible non‐causal explanations for this difference.^1^ The first of these that may be applicable here is the possibility that the NSWCCR Registry more often fails to link a woman's death to the record of her cancer registration than the WMNCRS, leading to apparently inflated survival. This explanation is very unlikely to apply in this younger age group during this period of time – these are young women among whom death is a relatively rare event, and who were followed up during a period of reliable death registration. The second possible explanation is that a higher proportion of *in situ* tumours registered in New South Wales were misclassified as invasive breast cancers than in the West Midlands. Again, we do not consider that this could be an explanation for the differences observed in this study, since >99% of the tumours analysed were microscopically verified and *in situ* cancers were excluded. We have also previously considered the accuracy and consistency of date of diagnosis as a potential mechanism by which survival in New South Wales might be extended relative to West Midlands. Again, however, this explanation has very little credibility here, since the date of diagnosis is established in the same manner for both screen‐detected and non‐screen‐detected women.

## Potential Explanations

Examining possible explanations operating before diagnosis, the differences in breast cancer survival between West Midlands and New South Wales could arise from (a) greater delays in diagnosis in West Midlands, (b) longer waiting times for hospital consultation for non‐screen‐detected cancers or (c) less effective screening in West Midlands than in New South Wales. The fact that differences persist after adjustment for extent of disease at diagnosis does not support any of these explanations, however. It is theoretically possible that residual confounding may partially account for this lack of explanatory power. Residual confounding may have arisen due to the fact that the screening interval for women in West Midlands is longer than in New South Wales, combined with a tendency for the accuracy of the ‘extent of disease’ variable for non‐screen‐detected cancers to be lower in West Midlands. Together, this would imply that within each stage grouping, the true (unknown) stage of disease is more advanced in West Midlands than in New South Wales (stage migration).^29^ This would have the effect of better extent‐adjusted survival in New South Wales. Although this explanation is possible, we consider that in the context of this study it is not very likely. This is because two mechanisms would both need to apply: delays for non‐screen‐detected cancers matched with less effective screening for screen‐detected cancers leading to differences in extent of a similar magnitude in both groups. At the very least, the fact that there is a significant difference in survival amongst women with screen‐detected disease in the two regions refutes the hypothesis that international differences are entirely due to practitioner delay in referral (since all these women were diagnosed through routine screening) or differences in patient delay in seeking medical diagnosis following the detection of breast cancer symptoms.

These findings thus tend to refute the idea that breast cancers in West Midlands and New South Wales are very different at the point of diagnosis, but are subsequently treated with similar effectiveness. We consider a much more likely explanation for our findings is that the tumours themselves are not substantially different, but that something different happens once the woman is diagnosed. This is of greater concern: we have examined a group of young women with predominantly localised disease. Almost all of them would have had treatment with curative intent.

Treatment may vary with comorbidity, and is subject to patient compliance, but there is no particular reason to assume that these would persistently differ between New South Wales and West Midlands, particularly in this age group. The alternative explanation is that the treatment in West Midlands is not as effective as those in New South Wales leading to poorer stage‐specific survival.^24^

## Conclusions

Although overall survival was high for this cohort of women, our data suggest that more than one in three breast cancer deaths within five years of diagnosis in the West Midlands would be avoidable if five‐year survival were the same as in New South Wales. The women we analysed here are relatively young. They are therefore less likely to be suffering from other serious illnesses and more likely to be economically and socially active. In order to improve the prognosis for women diagnosed with breast cancer in the UK during their early 50s it is essential that we understand better the mechanisms that underlie these international differences in stage‐adjusted survival. Differences in the effectiveness of treatment are an important possibility and they now deserve to be examined with great care. It is not possible to dismiss differences in breast cancer survival between the UK and other countries such as Australia as artefactual.
